# Pharmacologic inhibition of RGD‐binding integrins ameliorates fibrosis and improves function following kidney injury

**DOI:** 10.14814/phy2.14329

**Published:** 2020-04-13

**Authors:** Jeannine Basta, Lynn Robbins, Lisa Stout, Michael J. Prinsen, David W. Griggs, Michael Rauchman

**Affiliations:** ^1^ Department of Medicine Division of Nephrology Washington University School of Medicine Saint Louis Missouri; ^2^ VA St. Louis Health Care System Saint Louis Missouri; ^3^ Department of Biochemistry and Molecular Biophysics Washington University School of Medicine Saint Louis Missouri; ^4^ Department of Molecular Microbiology and Immunology Saint Louis University Saint Louis Missouri

**Keywords:** fibrosis, kidney injury, RGD integrin, TGF‐beta

## Abstract

Fibrosis is a final common pathway for many causes of progressive chronic kidney disease (CKD). Arginine–glycine–aspartic acid (RGD)‐binding integrins are important mediators of the pro‐fibrotic response by activating latent TGF‐β at sites of injury and by providing myofibroblasts information about the composition and stiffness of the extracellular matrix. Therefore, blockade of RGD‐binding integrins may have therapeutic potential for CKD. To test this idea, we used small‐molecule peptidomimetics that potently inhibit a subset of RGD‐binding integrins in a murine model of kidney fibrosis. Acute kidney injury leading to fibrosis was induced by administration of aristolochic acid. Continuous subcutaneous administration of CWHM‐12, an RGD integrin antagonist, for 28 days improved kidney function as measured by serum creatinine. CWHM‐12 significantly reduced *Collagen 1* (*Col1a1*) mRNA expression and scar collagen deposition in the kidney. Protein and gene expression markers of activated myofibroblasts, a major source of extracellular matrix deposition in kidney fibrosis, were diminished by treatment. RNA sequencing revealed that inhibition of RGD integrins influenced multiple pathways that determine the outcome of the response to injury and of repair processes. A second RGD integrin antagonist, CWHM‐680, administered once daily by oral gavage was also effective in ameliorating fibrosis. We conclude that targeting RGD integrins with such small‐molecule antagonists is a promising therapeutic approach in fibrotic kidney disease.

## Graphical abstract

Fibrosis is a final common pathway for many causes of progressive chronic kidney disease (CKD). Arginine–glycine–aspartic acid (RGD)‐binding integrins are important mediators of the pro‐fibrotic response by activating latent TGF‐β at sites of injury and by providing myofibroblasts information about the composition and stiffness of the extracellular matrix. Targeting RGD integrins with such small‐molecule antagonists is a promising therapeutic approach in fibrotic kidney disease.

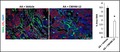

## 1 INTRODUCTION

Chronic kidney disease (CKD) affects ~15% of the population. In many affected individuals, there is progression to end‐stage kidney disease (ESKD) leading to kidney failure and a need for dialysis and transplantation. Although a broad range of insults can initiate kidney injury, interstitial fibrosis is a final common pathologic mechanism of most causes of progressive CKD. Studies in animal models and humans support the conclusion that the degree of interstitial fibrosis and tubular atrophy is strongly correlated with the severity of CKD in diverse forms of kidney disease (Klahr & Morrissey, 2002; Lee, Kim, & Choi, 2015), even after adjusting for eGFR, proteinuria, and clinicopathological diagnosis (Srivastava et al., 2018). Thus, targeting pro‐fibrotic pathways is an important strategy to slow the progression of CKD. However, with the exception of renin–angiotensin (RAAS) blockers for treatment of glomerular diseases, there are currently no specific therapies to ameliorate fibrosis and slow the decline in renal function. Moreover, while RAAS blockers slow the progression of proteinuric renal diseases, they do not arrest the disease or prevent the progression to end‐stage kidney failure. Therefore, the development of new therapies for CKD is an important priority in the field.

Acute kidney injury (AKI) affects ~8%–16% of patients admitted to a hospital (Sawhney & Fraser, 2017). Epidemiological studies have documented an apparent rise in the population incidence of AKI and acute dialysis. This is an important public health concern because in addition to the high mortality associated with AKI, recent studies point to the serious long‐term sequelae of CKD and dependence on renal replacement therapy for AKI survivors. AKI is a significant risk factor for postrecovery fibrosis and progressive CKD (Chawla & Kimmel, 2012). AKI is associated with an 8.8‐fold increased risk for CKD and a 3.3‐fold increase in ESKD (Coca, Singanamala, & Parikh, 2012). It may account for up to 20% of the incidence of new dialysis patients (Coca, 2010; Coca et al., 2012). However, there are no known effective therapies to prevent development of fibrosis after AKI. The 13th Acute Dialysis Quality Initiative concluded that research on the best treatment strategies for targeting progression after AKI is a major priority (Basile et al., 2016). Because AKI typically occurs in the hospital setting, there is a potential to intervene during the acute or early recovery phase to prevent the development of fibrosis and CKD.

A large body of evidence supports the conclusion that TGF‐β plays a critical role in promoting kidney fibrosis, making it a prime therapeutic target. However, because TGF‐β has a broad range of functions in both normal human physiological and pathological processes, developing effective therapies that target this cytokine has proved very challenging. Global inhibition of TGF‐β is associated with deleterious effects, especially with chronic fibrotic diseases such as CKD that require long‐term treatment (Nishimura, 2009; Sureshbabu, Muhsin, & Choi, 2016). Moreover, global blockade of TGF‐β may inhibit other actions, such as its anti‐inflammatory properties that promote proper repair (Sureshbabu et al., 2016). A more promising approach is to selectively target TGF‐β in its extracellular microenvironment in the injured tissue and prevent the transition from an inactive (latent) to an active state (Bouchie & DeFrancesco, 2015). Although TGF‐β activation can occur through various mechanisms, in the context of organ injury states that lead to fibrosis it is now well‐established that locally upregulated integrins are the primary mediators of latent TGF‐β activation (Henderson et al., 2013; Nishimura, 2009; Worthington, Klementowicz, & Travis, 2011). All five integrins incorporating the αv subunit (αvβ1, αvβ3, αvβ5, αvβ6, and αvβ8) have been shown in vitro to bind and activate latent TGF‐β through the amino acid sequence Arg‐Gly‐Asp (RGD) in the latency‐associated peptide (Asano, Ihn, et al., 2005; Asano, Yamane, Jinnin, Mimura, & Tamaki, 2005; Mu et al., 2002; Munger et al., 1999; Reed et al., 2015). Once activated, TGF‐β signaling through its own receptor induces the expression of many of the same integrin subunits (Honda, Yoshida, & Munakata, 2010; Zambruno et al., 1995), thereby establishing a local positive amplification loop that can perpetuate fibrosis until the loop is disrupted by drugs or processes that interfere with TGF‐β activation. Unlike the global inhibition produced by agents that target already‐activated TGF‐β (e.g., fresolimumab and galunisertib), integrin antagonism would exploit the body's natural mechanism of locally boosting TGF‐β at sites of tissue remodeling. Dampening the activity of these induced activated integrins is hypothesized to facilitate return to the homeostatic state without the systemic side effects that have been observed with indiscriminate global anti‐TGF‐β therapy. Independent of their role in TGF‐β activation, RGD‐binding integrins are also critical transducers of biomechanical force allowing cells to respond to matrix stiffness by promoting myofibroblast differentiation, migration, and survival (Fiore et al., 2018; Lampi & Reinhart‐King, 2018; Santos & Lagares, 2018). Interference with these signals thus may alter myofibroblast numbers and/or functions in an incipient or ongoing disease state.

To test the potential therapeutic utility of combined targeting of the subset of RGD‐binding integrins implicated in pro‐fibrotic molecular and cellular processes, we employed stable peptidomimetic small‐molecule inhibitors of these integrins in an established nephrotoxic model of kidney fibrosis.

## 2 METHODS

### 2.1 Compound synthesis and integrin function assays

The RGD peptidomimetic CWHM‐12 was synthesized as described in detail previously (Henderson et al., 2013). A close structural analog of this compound with improved oral bioavailability, CWHM‐680, was also synthesized at Saint Louis University. Potency for the compounds in blocking cell attachment mediated by integrins αvβ3, αvβ5, and αvβ6 was measured as previously described (Henderson et al., 2013). Potency for blocking cell attachment mediated by integrins αvβ1, αvβ8, and α8β1 was measured using modifications of this method which are briefly summarized as follows: To assess CWHM‐12 and CWHM‐680 effects on cellular αvβ1 function, we varied its concentration in an assay measuring binding of HEK‐293 cells, which naturally express this integrin (Nagarajan et al., 2007), to the surface of 96‐well plates coated with purified recombinant human TGFβ‐1 latency‐associated peptide (R&D Systems). To assess the effect on cellular αvβ8 function, we performed the assay with the same LAP ligand but with HEK‐293 cells which had been stably transfected to overexpress this integrin. To assess the effect on cellular α8β1 function, we performed the assay using HEK‐293 cells, which had been stably transfected to overexpress this integrin and used purified recombinant mouse nephronectin as the immobilized ligand (R&D Systems). To assess the effect on cellular α5β1 function, we performed the assay using K562 cells, which naturally express this integrin and used purified human plasma fibronectin (Calbiochem/EMD Biosciences) as the immobilized ligand. For all assays except α8β1, the optimal ligand concentration was defined as that providing maximum inhibition of the relevant cell binding by known specific function‐neutralizing antibodies while retaining strong binding in the presence of isotype‐matched negative control antibodies. Because no validated α8‐specific neutralizing antibodies are commercially available, optimization of ligand coating was performed by comparison of attachment of the α8β1‐overexpressing cells to the parental nontransfected cells.

### 2.2 Kidney injury model

Alzet osmotic minipumps (Cupertino, CA) were implanted subcutaneously in 8‐ to 10‐week‐old wild‐type male ICR outbred mice (Envigo) one day prior to induction of kidney injury to deliver vehicle (DMSO/H_2_O 1:1) or CWHM‐12 at a dose of 100 mg/kg per day. A single 5 mg/kg dose of aristolochic acid I sodium salt (Sigma‐Aldrich, A9451) in PBS was administered intraperitoneally to induce kidney injury, and mice were monitored daily for 27 days thereafter. Control (uninjured) mice were injected with an equal volume of PBS. Blood was obtained by maxillary vein puncture on days 0, 5 (peak injury), and 28 (study endpoint). For testing CWHM‐680, oral gavage (100 mg/kg per day) was started one day prior to injection of aristolochic acid I sodium salt and continued once daily until the study endpoint. Blood was obtained at days 0, 7, and 23 (study endpoint). Animals that did not survive until the end of the study were not included in serum creatinine analysis. Serum creatinine was measured by liquid chromatography–mass spectrometry (LC–MS/MS) at the University of Alabama O’Brien Center Bioanalytical Core. CWHM‐12 and CWHM‐680 concentrations were measured in plasma samples by liquid chromatography–tandem mass spectrometry (LC/MS/MS) using compound spiked into control plasma as a standard. All experiments were performed under protocols approved by the Institutional Animal Care and Use Committee at St. Louis University.

### 2.3 Tissue preparation, histology, and immunofluorescence

Harvested kidneys were bisected and harvested for RNA and protein, and fixed in 4% PFA for antibody staining and histological staining with Sirius red or Masson's trichrome. Paraffin sections (4 µm) were stained with Sirius red or Masson trichrome and imaged with polarized light and bright‐field illumination. For immunofluorescence, 7‐μm frozen sections were washed with ice‐cold 100% methanol, boiled in 10 mM citric acid (pH 6) for 20 min, and incubated with primary antibodies against αSMA (Millipore), PDGFR‐β (gift from B. Stallcup), or with biotin‐LTL (Vector Laboratories). Reactivity was detected using fluorescently labeled secondary antibodies. Sections were counterstained with DAPI (Sigma‐Aldrich), mounted in Mowiol 4–88 (Poly Sciences), and digital images acquired using a Leica DM5000B epifluorescence microscope and Leica DFC365FX camera. To quantify percent area stained for Sirius red, PDGFR‐β, and α‐SMA, we analyzed images from each of at least 4 biological replicates by batch processing using a macro created in Image J. Images were converted to 8‐bit grayscale, the threshold adjusted, and percent area measured. To quantify intact proximal tubules with brush borders, LTL‐positive tubules were counted by an observer blinded to the experimental conditions using Image J.

### 2.4 Quantitative PCR

RNA was extracted using the RNeasy Plus Mini Kit (Qiagen). cDNA was prepared using the High‐Capacity RNA‐to‐cDNA Kit (Applied Biosystems). qRT‐PCR was performed using a Quant Studio 3 (Applied Biosystems) Thermocycler and SYBR Green PCR Master Mix (Life Technologies). The following primers were used for qRT‐PCR: *Col1a1* 5′‐ATGTTCAGCTTTGTGGACCTCC‐3′ and 5′‐CAAGCATACCTCGGGTTTCC‐3′, *Col3a1* 5′‐GCGAGCGGCTGAGTTTTATG‐3′ and 5′‐TAGGACTGACCAAGGTGGCT‐3′, *Acta2* 5′‐ATCTGGCACCACTCTTTCTATAACG‐3′ and 5′‐CAGTTGTACGTCCAGAGGCA‐3′, *Tgfb1* 5′‐CAACAATTCCTGGCGTTACC‐3′ and 5′‐AGCCCTGTATTCCGTCTCCT‐3′, *Tgfb2* 5′‐CAAAACCCCAAAGCCAGAGTG‐3′ and 5′‐TCACGTCGAAGGAGAGCCAT‐3′, *Tgfb3* 5′‐GCACTTTACAACAGCACCCG‐3′ and 5′‐ACTCTGCCCGGAACAGATTG‐ 3′, *Fn1* 5′‐GCTTTAAGCTCACATGCCAGT‐3′ and 5′‐GAGGCATGTGCAGCTCATC‐3′, *Foxd1* 5′‐GTTTAGCTCAGAGGGTCCATCTAT‐3′ and 5′‐AGTGCCAAGACAGAGCGACT‐3′, *Pdgrfb* 5′‐AACTGTCACCCACACCCTTG‐3′ and 5′‐ACCACCACTTTGAAGGGCAA‐3′, *Mmp2* 5′‐GATAACCTGGATGCCGTCGT‐3′ and 5′‐TGGTGTGCAGCGATGAAGAT‐3′, *Ctgf* 5′‐AGAGTGGAGCGCCTGTTCTA‐3′ and 5′‐GGCTTGGCGATTTTAGGTGTC‐3′, *Shroom3* 5′‐AATTTGGGGAGACACAGCCT‐3′ and 5′‐GCTCCGCCTCAGATAAGCAT‐3′, *Itga5* 5′‐ATCCAGTGCACCACCATTCA‐3′ and 5′‐TCCGAACCACTGCAAGGAC‐3′, and *Itgb5* 5′‐CACCCAAAATGTGCCTGGTG‐3′ and 5′‐AGAGGTAGGTTCCGGAGGAC‐3′. Real‐time reactions were performed in triplicate, and relative expression was calculated using the delta CT method and normalized to *Gapdh* 5′‐AGGTCGGTGTGAACGGATTTG‐3′ and 5′‐TGTAGACCATGTAGTTGAGGTCA‐3′ or *Hprt1* 5′‐TCAGTCAACGGGGGACATAAA‐3′ and 5′‐GGGGCTGTACTGCTTAACCAG‐3′ control transcripts (Kiefer, Robbins, & Rauchman, 2012).

### 2.5 RNA sequencing

Total RNA was isolated from four kidneys from each of the three study groups using the RNeasy Plus Mini Kit (Qiagen). The groups were as follows: (a) aristolochic acid plus vehicle, (b) aristolochic acid plus CWHM‐12, and (c) PBS plus vehicle. After ribosomal depletion, we constructed barcoded sequencing libraries using the Ion Total RNA‐seq v2 kits (Life Technologies) according to the manufacturer's instructions. Sequencing was performed on an Ion Torrent Proton with mean read lengths of 85–110 nucleotides, and reads were aligned to the mouse mm10 genome using the TMAP aligner map4 algorithm. Soft clipping at both 5′ and 3′ ends of the reads was permitted during alignment to accommodate spliced reads, with a minimum seed length of 20 nucleotides. Genome‐wide strand‐specific nucleotide coverages were calculated from the aligned bam files for each sample using the “genomecoveragebed” program in BEDTools (Quinlan & Hall, 2010), and the nucleotide coverage for all nonredundant exons for each gene was summed using custom R scripts (http://www.Rproject.org). Normalization factors were calculated by averaging the total exon coverage for all replicates and dividing this average by the total exon coverage for each individual sample. The total coverage for each gene in each replicate was then multiplied by these factors after adding an offset of 1 to each gene to preclude division by 0 in subsequent calculations. The averages and *p* values of the coverage values for all genes in the individual groups were calculated using Microsoft Excel, using a two‐tailed *t*‐test. The expression values for each gene are the normalized strand‐specific total nucleotide coverage for each gene. The complete data set can be accessed at https://figshare.com/s/54c7f78b4523873e9b00.

### 2.6 Western blotting

Protein lysates were made by homogenizing kidney tissue in cold 50 mM Tris pH 7.5, 150 mM NaCl, 1% Triton X‐100, and 200 mM sucrose with protease inhibitors using a Tissue Tearor. Protein lysates were run on SDS‐PAGE gels and proteins detected using Collagen 1 (Proteintech, cat# 14695‐1‐AP, 1:3,000) and HDAC2 (Abcam, cat# ab32117, 1:3,000) primary antibodies and peroxidase‐labeled goat anti‐rabbit secondary antibody (Sigma‐Aldrich, cat# A0545, 1:10,000). Signal was developed with SuperSignal West Dura (Thermo Fisher). Collagen 1 densitometry was performed using Image J and normalized to HDAC2.

### 2.7 Statistical analysis

Data are presented as the mean ± *SEM*. Statistical analysis was performed using a one‐tailed unpaired Student's *t*‐test or ANOVA followed by multiple comparisons analysis with Tukey's correction (GraphPad Prism). Statistical analysis for each experiment is noted in the figure legend. We considered differences with a *p* value of .05 or less to be statistically significant.

## 3 RESULTS

### 3.1 CWHM‐12 inhibits cell–ligand interactions mediated by RGD integrins

The small‐molecule RGD peptidomimetic compound CWHM‐12 has been shown previously to inhibit cell–ligand interactions mediated by αvβ3, αvβ5, and αvβ6, and the interactions of biochemically purified integrins αvβ1 and αvβ8 with their respective ligands (Henderson et al., 2013). We have now determined the potency of this compound against these and additional RGD‐binding integrins entirely using cell‐based assays (Table 1). These results show particularly strong potency (<1 nM) for αvβ1, αvβ3, and αvβ6, with varying lesser activities for the other tested RGD‐binding integrins. As previously reported, CWHM‐12 has no significant activity (>5 μ M) against integrin αIIbβ3, which is essential for platelet aggregation, nor does it affect ligand binding by non‐RGD‐binding integrins (Henderson et al., 2013).

**Table 1:** Potency of test compounds in inhibiting integrin‐mediated cell attachment to ligands^a^

	αvβ1	αvβ3	αvβ5	αvβ6	αvβ8	α5β1	α8β1
CWHM−12	0.29 ± 0.14	0.47 ± 0.45	47 ± 26	0.31 ± 0.14	5.8 ± 2.3	8.0 ± 3.9	35 ± 16
CWHM−680	1.24 ± 0.12	1.18 ± 0.74	4.5 ± 2.2	0.39 ± 0.17	18.6 ± 5.0	100 ± 37	424 ± 295

a Values represent the mean IC_50_ (nM) and standard deviation determined from at least 3 independent assays.

### 3.2 CWHM‐12 ameliorates kidney function and fibrosis in a model of nephrotoxic injury

**Figure 1:**
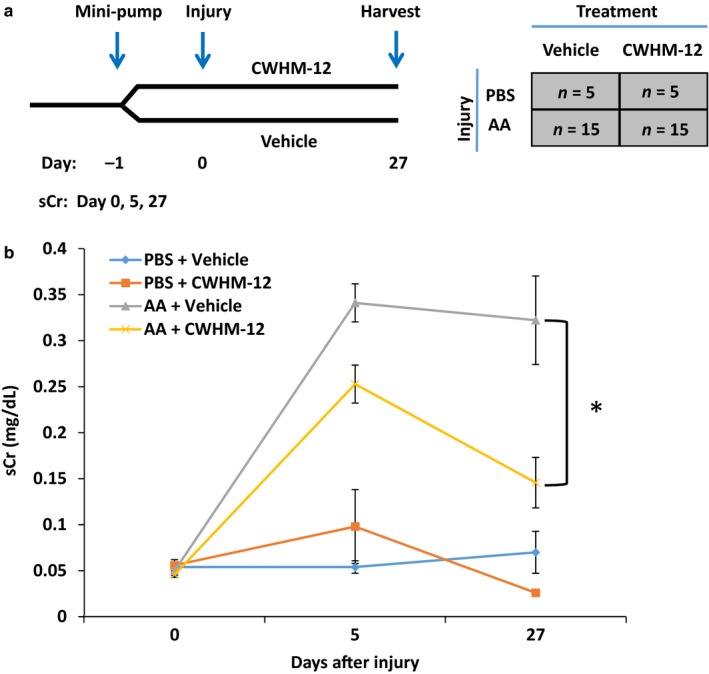
(a) Schematic of study design. Osmotic minipumps were inserted one day prior (day −1) to administration of aristolochic acid (AA, day 0) to induce kidney injury. Animals were randomized to receive CWHM‐12 at 100 mg kg^−1^ day^−1^ or vehicle for 27 days after AA. (b) Serum creatinine (sCr mg/dl) in injured (AA) and uninjured (PBS) animals that received vehicle or CWHM‐12. sCr was significantly decreased at day 27 in AA‐treated animals that received active drug (CWHM‐12) compared with vehicle. In uninjured (PBS) control animals, sCr was not affected by CWHM‐12. PBS + vehicle, *n* = 5; PBS + CWHM‐12, *n* = 5; AA + vehicle, *n* = 9; AA + CWHM‐12, *n* = 7. In (b), data were analyzed by two‐way ANOVA followed by multiple‐group comparison analysis with Tukey's correction, *p < .05

We examined the effect of CWHM‐12 treatment on kidney fibrosis in a nephrotoxic model of acute kidney injury. Mice received an intraperitoneal injection of aristolochic acid (AA, 5 mg/kg body weight) to induce injury and were monitored for 27 days. CWHM‐12 (100 mg/kg body weight) or vehicle control was delivered subcutaneously by Alzet minipump beginning one day prior to induction of kidney injury (Figure 1a). Determination of compound levels from blood samples collected on various days showed the mean steady plasma drug concentration was approximately 5 µg/ml (Table S1 https://figshare.com/s/54c7f78b4523873e9b00), similar to that measured in previous studies in which this dosing regimen was used to evaluate efficacy in other models (Henderson et al., 2013; Ulmasov et al., 2016). At day 5 after AA injection, serum creatinine was significantly increased in both control and CWHM‐12‐treated injured animals (Figure 1b), consistent with previous studies showing acute injury due to AA (Hirsch et al., 2015; Novitskaya et al., 2014; Yang, Besschetnova, Brooks, Shah, & Bonventre, 2010). At day 27, serum creatinine was reduced from its peak value at day 5 in CWHM‐12‐treated animals but not in vehicle‐treated injured animals (0.15 mg/dl ± 0.027 vs. 0.32 mg/dl ± 0.048, *p* = .0334). We conclude that CWHM‐12 treatment protects kidney function in a model of nephrotoxic injury.

**Figure 2:**
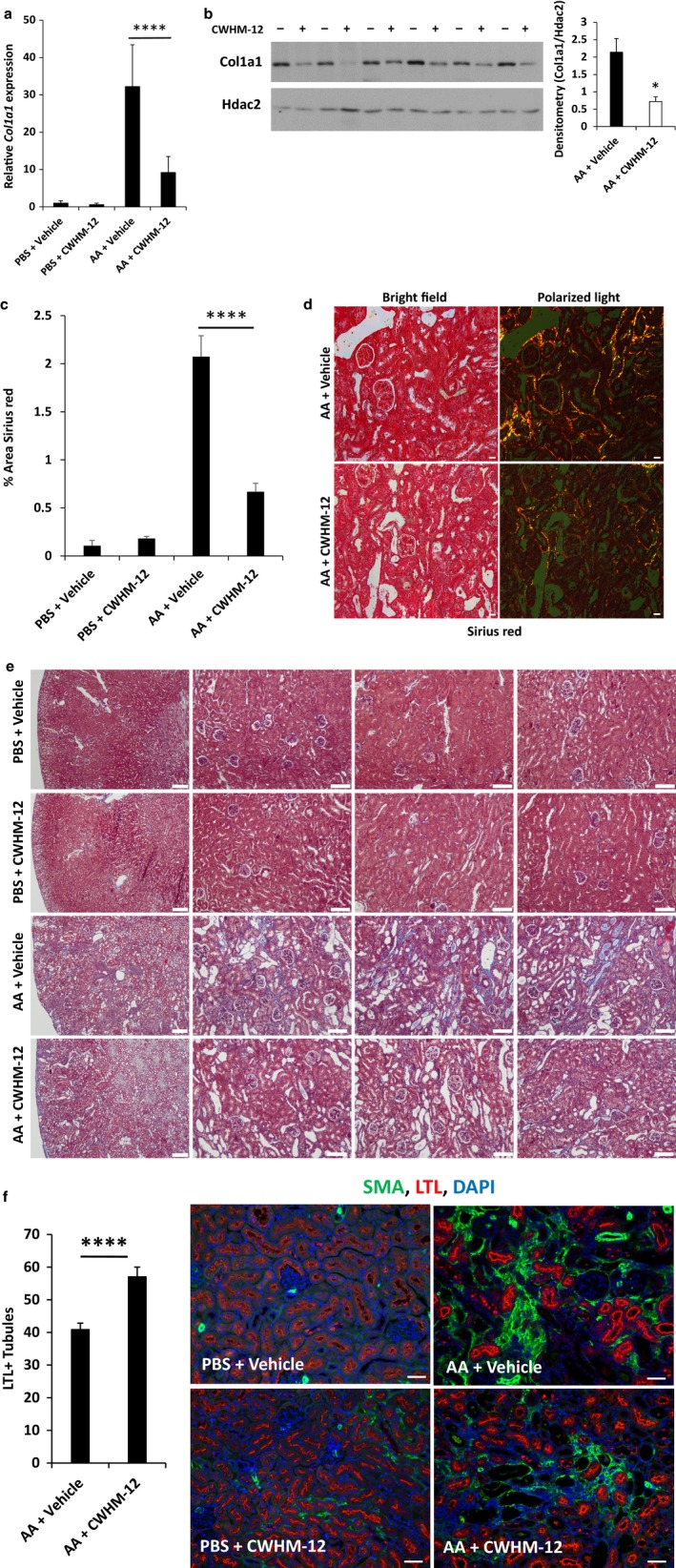
(a) *Collagen 1* (*Col1a1*) mRNA expression was determined by real‐time PCR in injured and uninjured control animals treated with CWHM‐12 or vehicle. Relative to vehicle‐treated animals, the induction of *Col1a1* mRNA expression was attenuated in AA‐injured animals treated with CWHM‐12 (32‐fold vs. 9‐fold, compared with controls). AA + vehicle *n* = 9, AA + CWHM‐12, *n* = 7. (b) Collagen 1 protein was detected by Western blot in animals treated with CWHM‐12 or vehicle. Quantitation by densitometry revealed a threefold reduction in Collagen 1 (0.719 ± 0.13 vs. 2.14 ± 0.39 arbitrary units, *p* = .00345, *n* = 6 for each group) with CWHM‐12 treatment. (c) Quantitation of collagen deposition by analysis of percent area kidney sections stained by Sirius red. Relative to vehicle‐treated animals, the percent area stained by Sirius red was reduced by 68% in AA‐injured animals that were treated with CWHM‐12. *n* = 5 for each AA group, with at least 40 images analyzed for each group, ****p <.0001. (d) Bright‐field and polarized light images are shown for Sirius red staining of kidney tissue. Quantitation was performed using polarized light, scale bar = 25µm. (e) Masson trichrome staining revealed a reduction in collagen staining in CWHM‐12 compared with vehicle‐treated animals. One low power image (scale bar = 200µm) shown for each group and 3 different images from 3 different kidneys for each group shown in higher power (scale bar = 100 µm). (f) LTL staining showed significantly increased proximal tubules with intact brush borders in CWHM‐12‐treated animals compared with vehicle‐treated. At least 8 images from each biological replicate were counted from each group, *n* = 5 for each group, ****p < .0001, scale bar = 50µm. For a and c, data were analyzed by ANOVA followed by multiple‐group comparison's analysis with Tukey's correction. For b and f, data were analyzed by unpaired *t*‐test

Aristolochic acid administration is a recognized cause of postacute kidney injury fibrosis in mice and humans (Novitskaya et al., 2014; Yang et al., 2010). In rodent models, fibrosis was apparent within 14 days of AA administration and was progressively worse at day 28. We next examined whether the improvement in renal function at 27 days in CWHM‐12‐treated animals correlated with a reduction in kidney fibrosis. To determine drug effect on kidney fibrosis, we measured pro‐fibrotic gene expression and performed staining on kidneys to assess interstitial collagen deposition. Collagen 1 (*Col1a1*) mRNA expression was increased 32‐fold by qRT‐PCR in AA‐injected animals treated with vehicle compared with uninjured animals. In contrast, in injured animals treated with CWHM‐12, *Col1a1* expression was increased just 9‐fold. Collagen 1 protein expression was similarly reduced threefold as determined by Western blot in CWHM‐12 compared with vehicle treatment. Thus, CWHM‐12 significantly attenuated upregulation of *Col1a1* (Figure 2a and b), a major component of ECM deposition in organ fibrosis, at the mRNA and protein level. Sirius red staining to assess scar collagen deposition in injured kidneys revealed a 68% (*p* < .0001) reduction in the percentage of area stained in CWHM‐12‐treated animals compared with vehicle‐treated (Figure 2c and d). Masson trichrome staining showed reduced collagen deposition in drug versus vehicle‐treated animals (Figure 2e). The reduction in fibrosis and improved kidney function in CWHM‐12‐treated animals suggested that repair or preservation of injured tubules would be enhanced by drug treatment. We quantitated the number of proximal tubules with intact brush borders based on LTL staining (Figure 2f). The number of intact proximal tubules was significantly greater in CWHM‐12‐treated kidneys at 27 days after AA induced injury compared with vehicle‐treated animals (57.1 ± 1.7 vs. 40.9 ± 1.6, *n* = 5 for each group, *p* < .0001). These results demonstrated that inhibition of RGD integrins preserved intact proximal tubules, and ameliorated kidney fibrosis and renal dysfunction in a model of nephrotoxic kidney injury. Because sCr was not significantly different at day 5, the protective effect at day 27 cannot be attributed to a difference in the severity of the initial injury.

**Figure 3:**
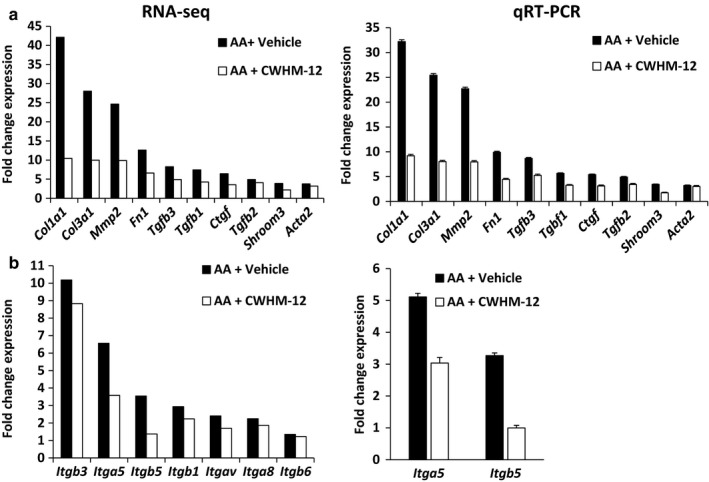
(a) Gene expression determined by RNA‐seq in injured (AA) animals exposed to vehicle or CWHM‐12. Expression of pro‐fibrotic cytokines *Tgfb1*, *Tgfb2, Tgfb3,* and *Ctgf*, genes encoding extracellular matrix proteins *Col1a1*, *Col3a1*, *Mmp2*, and *Fn1*, and the TGF‐β target gene *Shroom3* was reduced in CWHM‐12‐treated injured kidneys compared with vehicle‐treated. (*p* values are shown in Table 2). The expression level of pro‐fibrotic genes by qRT‐PCR was very similar to the RNA‐seq. (b) Expression of genes encoding RGD integrins was induced by injury. CWHM‐12 treatment significantly reduced the expression of all the RGD integrin genes, but only *Itgb5* reached statistical significance. RNA‐seq was performed on 4 biological replicates for each group. *Itga5* and *Itgb5* expression changes by qRT‐PCR were in agreement with RNA‐seq. For qRT‐PCR in a and b, data were analyzed by unpaired *t*‐test. All qRT‐PCR comparisons reached significance except for *Acta2*

To comprehensively analyze the effect of CWHM‐12 on pro‐fibrotic gene expression, we compared mRNA levels in kidneys of AA‐injured animals treated with vehicle and CWHM‐12 by RNA sequencing (RNA‐seq). We also analyzed the mRNA expression in vehicle‐treated uninjured animals. Injury‐induced expression of multiple pro‐fibrotic genes was significantly reduced in injured mice treated with CWHM‐12 compared with vehicle (*p* < .05, *n* = 3 biological replicates). This includes components of the extracellular matrix (ECM) (collagens, fibronectin, matrix metallopeptidases) and pro‐fibrotic cytokines, TGF‐β1‐3, and CTGF (Figure 3a and Table 2). *Shroom3* is a TGFβ‐1 target gene that promotes kidney fibrosis (Menon et al., 2015). In humans, an intronic SNP that conferred increased expression of this gene is associated with CKD in GWAS and with fibrosis in kidney allografts (Kottgen et al., 2009, 2010; Menon et al., 2015). Injury‐induced expression of *Shroom3* was attenuated by CWHM‐12 in injured kidneys (Figure 3a and Table 2). We performed qRT‐PCR to validate the gene expression changes determined by RNA‐seq for 12 pro‐fibrotic genes. Relative expression changes were in agreement with the RNA‐seq (Figure 3a and b).

**Table 2:** Fold change of profibrotic genes by RNA‐seq

Gene	AA + Vehicle	AA + CWHM−12	AA + CWHM−12/AA + Vehicle	*p* value
*Col1a1*	42.1	10.4	0.2480	.0013
*Col3a1*	28.0	10.0	0.3560	.0118
*Mmp2*	24.6	9.9	0.4013	.0116
*Fn1*	12.6	6.6	0.5243	.0949
*Tgfb3*	8.3	4.9	0.5937	.0738
*Tgfb1*	7.4	4.2	0.5715	.1163
*Ctgf*	6.4	3.5	0.5510	.1690
*Tgfb2*	4.9	4.1	0.8306	.6904
*Shroom3*	3.9	2.2	0.5633	.0871
*Acta2*	3.7	3.2	0.8473	.3541

TGF‐β is a central mediator of organ fibrosis in multiple tissues, including kidney. The ability of CWHM‐12 to inhibit the activation of latent TGF‐β in the ECM is predicted to be a major mechanism by which this compound ameliorated kidney fibrosis in our model. Consistent with this observation, pathway analysis of the RNA‐seq data showed that genes responding to TGF‐β signaling were significantly reduced (Figure 5, *p* = 10^–7^). However, in addition to promoting formation of myofibroblasts and pro‐fibrotic gene expression, TGF‐β also induces the expression of RGD integrin subunits and TGF‐β itself, thereby creating a self‐reinforcing loop (Honda & Munakata, 2010; Zambruno et al., 1995). Thus, treatment with CWHM‐12 would also be predicted to reduce RGD integrin and TGF‐β expression. As shown in Figure 3a, *Tgfb1‐3* mRNA expression was induced with injury as expected, but this was blunted in CWHM‐12‐treated mice. The RNA‐seq data also revealed that expression of the subunits that form RGD‐binding integrins increased in expression with injury, whereas the expression of this group was reduced by CWHM‐12 (Figure 3b). Thus, treatment with CWHM‐12 disrupted the amplification of pro‐fibrotic TGF‐β signaling mediated by the autoregulatory loop with RGD integrins.

**Figure 4:**
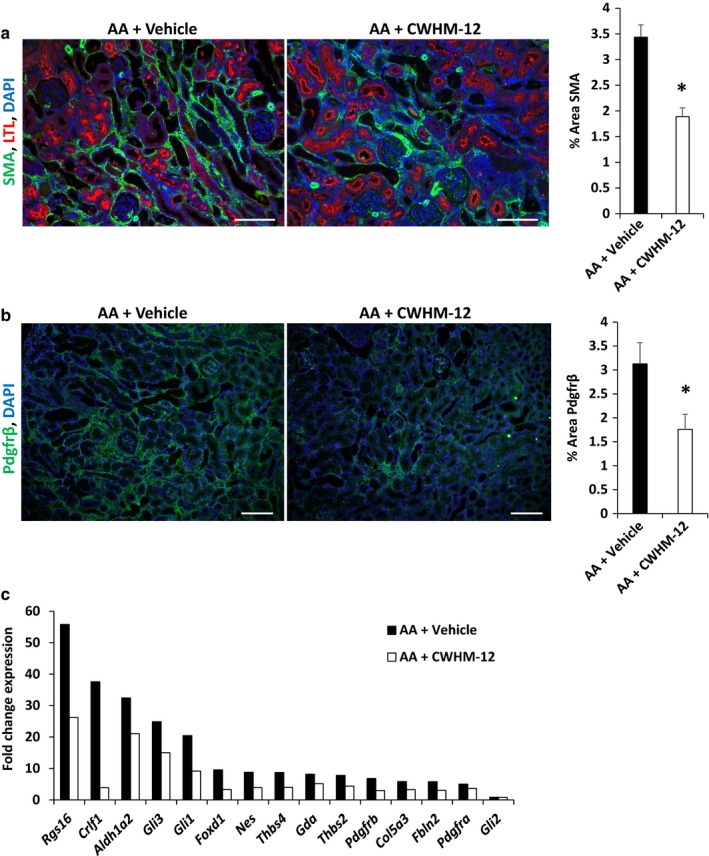
(a–b) Quantitation of percent area immunostained by PDGFR‐β and α‐SMA. Relative to vehicle‐treated animals, the percent area stained for PDGFR‐β (*p* = .008) and α‐SMA (*p* = 9.8 × 10^−7^) was reduced by 44% and 45%, respectively, in AA animals that were treated with CWHM‐12. For PDGFR‐β, *n* = 5 biological replicates with at least 20 images analyzed for each group; for α‐SMA, *n* = 5 biological replicates with at least 40 images analyzed for each group, scale bar = 100 µm. (c) Myofibroblast gene expression by RNA‐seq. Expression of multiple markers of activated myofibroblasts was induced by AA‐injured animals. CWHM‐12 significantly attenuated expression of these genes, with *Pdgfrb*, *Foxd1*, *Gli1*, and *Crfl1* reaching statistical significance at *p* < .05. RNA‐seq was performed on 4 biological replicates for each group. *p* values are shown in Table 3. For a and b, data were analyzed by unpaired *t*‐test

Myofibroblasts are the principal source of extracellular matrix deposition in kidney fibrosis. In AA‐injured kidneys, we found significant upregulation of several specific markers of activated myofibroblasts in the injured kidney that reflects their origin from perivascular mesenchymal cells (Grgic et al., 2014). Compared with vehicle, CWHM‐12 treatment markedly reduced *Gli1* (20.4‐ vs. 9.2‐fold), *Pdgfra* (5.0‐ vs. 3.7‐fold), *Pdgfrb* (6.8‐ vs. 3.0‐fold), *Foxd1* (9.6‐ vs. 3.3‐fold), and *Crlf1* (37.6‐ vs. 3.9‐fold) expression (Figure 4c and Table 3). We also examined myofibroblast formation by immunostaining kidneys with antibodies to αSMA and PDGFR‐β. We found that the percentage of area of expression for these proteins was reduced by 45% (*p* < 10**^–^** ^6^) for αSMA and by 44% for PDGFR‐β (*p* = .008) by CWHM‐12 treatment compared with vehicle‐treated animals (Figure 4a and b). We also compared our RNA‐seq data to that of Grgic et al. (2014) who performed transcriptional profiling of isolated myofibroblasts after unilateral ureteral obstruction (UUO) to identify genes induced in response to injury. We found that novel biomarkers of activated myofibroblasts identified by Grgic et al. were also significantly upregulated in our AA injury model (Figure 4c). Importantly, treatment with CWHM‐12 abrogated the induction of these genes, indicating that inhibition of RGD integrins prevented expansion or activation of myofibroblasts.

**Table 3:** Fold change of genes expressed in myofibroblasts by RNA‐seq

Gene	AA + Vehicle	AA + CWHM−12	AA + CWHM−12/AA + Vehicle	*p* value
*Rgs16*	55.80	26.19	0.4692	.16075
*Crlf1*	37.57	3.88	0.1032	.00004
*Aldh1a2*	32.44	21.06	0.6492	.12267
*Gli3*	24.87	15.02	0.6039	.23568
*Gli1*	20.44	9.17	0.4486	.00333
*Foxd1*	9.55	3.27	0.3427	.00829
*Nes*	8.77	3.91	0.4461	.05825
*Thbs4*	8.72	3.97	0.4558	.13873
*Gda*	8.17	5.15	0.6302	.20202
*Thbs2*	7.78	4.36	0.5603	.20064
*Pdgfrb*	6.79	2.98	0.4393	.03738
*Col5a3*	5.81	3.25	0.5587	.23788
*Fbln2*	5.78	3.04	0.5262	.07217
*Pdgfra*	5.02	3.66	0.7298	.26646
*Gli2*	0.87	0.78	0.8986	.81055

### 3.3 Multiple pro‐fibrotic pathways are attenuated and repair processes are enhanced by CWHM‐12

The RNA‐seq data also revealed that biomarkers implicated in kidney fibrosis and dysfunction were ameliorated by CWHM‐12 treatment. NGAL (*Lcn2*), a well‐established biomarker of acute and chronic kidney injury (Kiryluk et al., 2018), was induced 144‐fold in AA‐injured/vehicle‐treated relative to uninjured. In CWHM‐12‐treated/AA‐injured kidneys, NGAL expression decreased by 50% compared with vehicle‐treated injured mice. Reduced EGF expression has been associated with a higher percent area of interstitial fibrosis in human kidney tissue (Beckerman et al., 2017). In AA‐injured mice, *Egf* expression was reduced by 51% compared with uninjured mice, whereas *Egf* expression was increased twofold with CWHM‐12 treatment of injured animals. Multiple keratin (*Krt*) genes were recently identified as biomarkers of post‐AKI fibrosis (Cippà et al., 2018). We found that eight keratin genes (*Krt* 7, 8, 15, 18–20, 23, and 80) were upregulated with injury; in each case, the degree of upregulation was significantly reduced by ~15%–50% with CWHM‐12 administration.

*Sox9* is a transcriptional regulator that has a role in renal epithelial cell repair after acute injury; its expression was shown to be persistently elevated in the transition from AKI to CKD and fibrosis (Kang et al., 2016; Kumar et al., 2015; Li et al., 1864). Induction of *Sox9* (45‐fold) was decreased by 57% by CWHM‐12. *Sox9* has been linked to proliferating tubular epithelia after injury. In addition to *Sox9*, other genes associated with proliferation (*Fosl2*, *Myc*, *Ccnb1*, *Cdk1*) induced by injury displayed attenuated expression with CWHM‐12 compared with vehicle treatment. The cell cycle regulators *Ccnb1* and *Cdk1* were markedly upregulated in injured kidneys exposed to vehicle, 23‐fold and 12‐fold, respectively. CWHM‐12 treatment reduced expression to levels that were only 1.4‐fold and 1.9‐fold higher than in vehicle‐treated injured kidneys, suggesting that proliferation of tubular epithelia, myofibroblasts, or both was reduced by CWHM‐12. This is consistent with an attenuation of the injury response.

**Figure 5:**
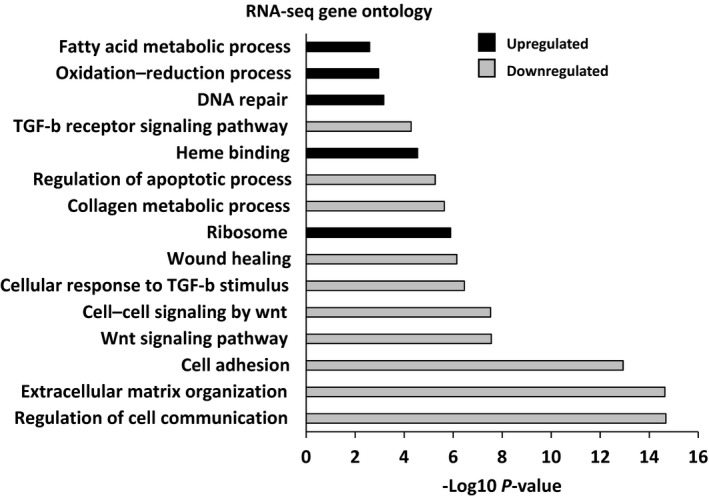
Gene ontology analysis of differentially expressed genes in injured animals treated with drug versus vehicle. Bar graph indicating p values for biological processes and pathways that were downregulated or upregulated by CWHM‐12 treatment compared with vehicle control in injured animals. Several pathways that promote fibrosis, such as TGF‐β and Wnt, were reduced by CWHM‐12 treatment, whereas pathways or processes associated with repair were upregulated

Pathway analysis of the RNA‐seq data revealed that CWHM‐12 affected biological processes that are consistent with reduced injury and enhanced repair. Cell adhesion and extracellular matrix organization are related pathways that were significantly upregulated with kidney injury and fibrosis in our data, and this is in agreement with (Cippà et al., 2018). However, when comparing drug and vehicle treatment we found that expression of genes in these same pathways was significantly attenuated by CWHM‐12 (Figure 5). Other well‐established pro‐fibrotic pathways were downregulated with CWHM‐12 treatment, including WNT and TGF‐β signaling (Figure 5). Mitochondrial dysfunction plays an important role in the pathogenesis of kidney injury via its critical role in cellular energetics and regulation of apoptosis. Compared with vehicle, CWHM‐12 treatment in animals exposed to AA showed gene expression changes that reflected improved oxidative phosphorylation and a reduction in the apoptotic pathway. Overall, the RNA‐seq data supported the conclusion that multiple deleterious pro‐fibrotic pathways were attenuated and processes indicative of adaptive repair or recovery were enhanced by treatment with this RGD integrin antagonist in nephrotoxic injury.

### 3.4 An orally dosed RGD peptidomimetic compound ameliorates kidney fibrosis

**Figure 6:**
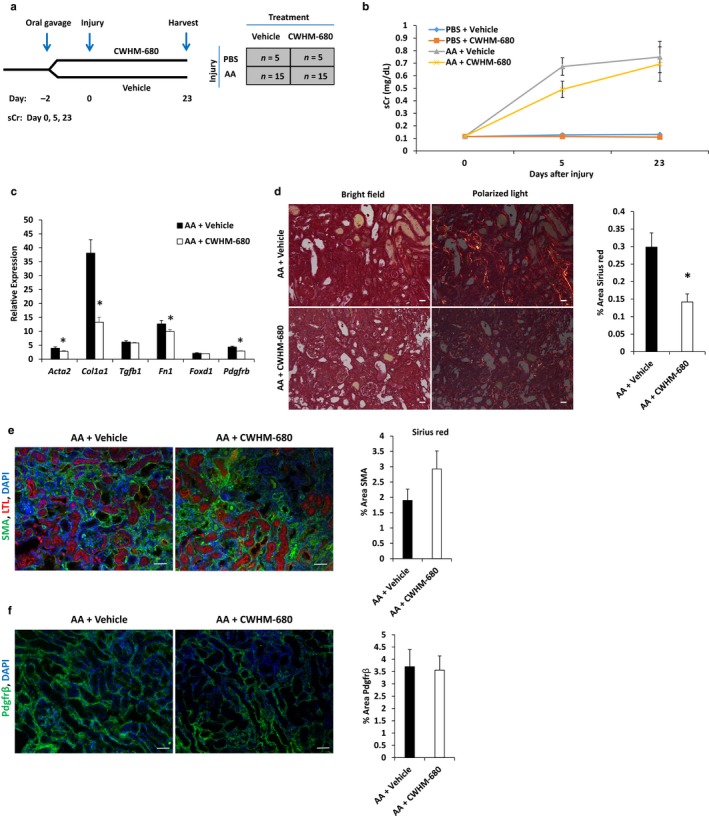
(a) Schematic of study design. Daily oral gavage of CWHM‐680 (100 mg kg^−1^ day^−1^) was started two days prior (day −2) to administration of aristolochic acid and continued until study endpoint (day 23). (b) Serum creatinine (sCr mg/dl) in injured (AA) and uninjured (PBS) animals that received vehicle or CWHM‐680. sCr was decreased at days 7 and 23 in AA‐treated animals that received active drug (CWHM‐680) compared with vehicle, although not statistically significantly. PBS + vehicle, *n* = 5; PBS + CWHM‐680, *n* = 4; AA + vehicle, *n* = 14; AA + CWHM‐680, *n* = 14. (c) mRNA expression was determined by real‐time PCR in injured and uninjured control animals treated with CWHM‐680 or vehicle. Relative to uninjured, vehicle‐treated animals, the induction of *Col1a1* mRNA expression was attenuated in AA‐injured animals treated with CWHM‐680 (38‐fold vs. 13‐fold, compared with controls. *p = 2.2 × 10^−5^). *Pdgfrb, Acta2,* and *Fn1* mRNA expression was also significantly reduced in injured animals treated with CWHM‐680 (*p < .05). AA + vehicle, *n* = 13; AA + CWHM‐680, *n* = 11. (d) Quantitation of collagen deposition by analysis of percent area of kidney sections stained by Sirius red. Relative to vehicle‐treated animals, the percent area stained by Sirius red was reduced by 52% in AA‐injured animals that were treated with CWHM‐680. *n* = 4 biological replicates with at least 36 images analyzed for each group, *p = .0005, scale bar = 25 µm. (e) Quantitation of percent area immunostained by PDGFR‐β and α‐SMA. Relative to vehicle‐treated animals, the percent area stained for PDGFR‐β and SMA was not attenuated in CWHM‐680‐treated injured animals compared with vehicle‐treated injured animals. *n* = 4 with at least 24 images analyzed for each group, scale bar = 50 µm. For b, data were analyzed by two‐way ANOVA followed by multiple comparison's analysis with Tukey's correction. For c, d, e, and f, data were analyzed by unpaired *t*‐test

To enhance the potential for translation to the clinic, we evaluated whether CWHM‐680, an analog of CWHM‐12 with significant oral bioavailability (*F* = 45% in mouse), could also reduce kidney fibrosis. As shown in Table 1, CWHM‐680 also potently inhibits the alpha v subfamily of integrins, though it is comparatively less potent against α5β1. We administered vehicle or CWHM‐680 (100 mg/kg body weight) once daily by oral gavage beginning two days prior to injury by administration of AA (Figure 6a). Average total compound concentrations measured from blood samples collected just prior to administration of the next scheduled dose on days 1, 7, and 23 were 0.385, 0.464, and 2.279 µg/ml, respectively (Table S2 https://figshare.com/s/54c7f78b4523873e9b00), in injured animals. These trough levels of CWHM‐680 were approximately 2–14 times lower than the steady‐state concentration measured in mice continuously infused with CWHM‐12. Although we did not find a reduction in sCr at the study endpoint (Figure 6b), several independent measures indicated that CWHM‐680‐treated animals developed less kidney fibrosis. *Col1a1* mRNA expression was reduced 3‐fold, and fibronectin was reduced by 22% with drug treatment compared with control animals (Figure 6c). Consistent with the reduction in scar collagen expression, we found that the percentage area staining for Sirius red was reduced by 52% (*p* = .0006, *n* = 4 for each group, Figure 6d). *Pdgfrb* mRNA expression was reduced by 33% and *Acta2* was reduced by 28% with CWHM‐680 treatment, indicating that the drug limited myofibroblast formation after injury (Figure 6c). However, in contrast to CWHM‐12, CWHM‐680 did not reduce the percent area of αSMA or Pdgfr‐β staining or *Tgfb* expression (Figure 6c and e–f), suggesting that the CWHM‐680 oral treatment regimen may have been less effective overall than the CWHM‐12 infusion method for several possible reasons (see Discussion).

## 4 DISCUSSION

TGF‐β signaling is a central mediator of fibrosis in multiple tissues. Consequently, targeting its activity has been the goal of many drug strategies. However, global targeting of TGF‐β in the clinic has been unsuccessful due to serious adverse effects stemming from its diverse roles in normal physiology. A more promising approach is to target integrin activators of TGF‐β which are locally upregulated at sites where fibrosis is being stimulated. All five members of the alpha v integrin family (αvβ1, αvβ3, αvβ5, αvβ6, and αvβ8) have been shown to be expressed at sites of injury either on damaged epithelial cells or on activated myofibroblasts. They all bind to the arginine–glycine–aspartic acid (RGD) motif of the latency‐associated peptide (LAP) and are capable of releasing biologically active TGF‐β that triggers a pro‐fibrotic signaling program (Henderson et al., 2013; Nishimura, 2009; Worthington et al., 2011).

Here, we report that blocking activation of TGF‐β with small‐molecule peptidomimetic inhibitor of a subset of RGD integrins reduced pro‐fibrotic gene expression, reduced kidney fibrosis, and ameliorated renal function in a model of nephrotoxicity. The steady‐state drug levels of CWHM‐12 we measured with continuous administration by minipump were very similar to those found to reduce fibrosis after pancreatic injury and likely represent a maximally effective dose (Ulmasov et al., 2016). In addition to ameliorating kidney fibrosis and renal impairment, it is significant that we did not observe obvious deleterious effects in CWHM‐12‐treated animals. This is consistent with data from clinical studies for cancer indications in which RGD integrin inhibitors with varying target selectivity profiles were found to be generally well‐tolerated and safe (Cirkel et al., 2016; Hariharan et al., 2007; O'Day et al., 2011). However, more detailed toxicology studies will be needed to ensure the safety of such compounds for treatment of kidney diseases. In addition to therapeutic efficacy, the route of administration is an important consideration for translating a therapy to the clinic for kidney fibrosis; an oral agent is preferred because long‐term treatment is required. We therefore tested an analog of CWHM‐12 with improved oral bioavailability in our nephrotoxicity model. CWHM‐680 was also effective in ameliorating kidney fibrosis, but it did not reduce sCr at the study endpoint of 23 days. The differing pharmacokinetic and pharmacodynamic profiles for the two compounds, one delivered by continuous infusion and the other with daily oral gavage, likely account for the overlapping but distinct endpoint outcomes of the two drugs. Moreover, we cannot exclude the possibility that their different potency for inhibition of α5β1 integrin may be a contributing factor (Table 1), as discussed below. While further studies are needed to optimize dosing of CWHM‐680 and test longer study endpoints, our data indicate that this oral agent was effective in ameliorating fibrosis.

Alpha v (αv) integrins are ubiquitously expressed in the adult kidney (Pozzi & Zent, 2013), so there is potential for functional redundancy when these receptors are activated during repair processes. The fact that most RGD integrins were upregulated with injury in our study (Figure 3b) further underscores the potential functional redundancy, thereby supporting the utility of an antagonist of multiple RGD integrins. In support of this idea, different αvβ integrin heterodimers have been implicated in kidney fibrosis. αvβ6 integrin is expressed on renal tubular epithelia and is upregulated in response to injury (Breuss et al., 1995; Ma et al., 2003). Genetic deletion of the integrin β6 subunit confers significant protection from development of fibrosis and activation of TGF‐β signaling in unilateral ureteral obstruction (Ma et al., 2003). In a model of nephrotoxic injury due to adenine, a small model inhibitor of αvβ1 ameliorated kidney dysfunction and fibrosis (Chang et al., 2017). Kidney myofibroblasts express several αv integrins (αvβ1, αvβ3, αvβ5) (Chang et al., 2017). Cell‐selective αv deletion from the myofibroblast lineage was protective against fibrosis in several organ injury models including kidney (Henderson et al., 2013). Our studies showed a significant reduction in the percent area stained for SMA and PDGFR‐β, protein markers of myofibroblasts, in CWHM‐12‐treated animals. We also found that myofibroblast gene expression was broadly attenuated by this compound. Together, these findings indicate that effects of RGD integrin antagonism on formation or activity of myofibroblasts are important for its protection against kidney fibrosis. There are some reports showing that loss of specific integrins may predispose mice to kidney fibrosis (Pozzi & Zent, 2013). For example, mice lacking α8 (i.e., α8β1) showed increased fibrosis after UUO (Hartner et al., 2012). Our data using a cell‐based assay of α8β1‐mediated nephronectin binding suggest CWHM‐12 may be only a weak inhibitor of this integrin. Since the compound has much stronger activity against other pro‐fibrotic integrins, this may have been sufficient to counteract this potentially opposing effect.

Beckerman et al. recently showed that global gene expression was a very sensitive indicator of fibrosis in human kidneys and this in turn was a strong determinant of renal function (Beckerman et al., 2017). Similarly, analysis of our RNA‐seq data revealed that RGD integrin blockade influenced many pathways linked to fibrosis and improved renal function in a model of nephrotoxicity. Consistent with the important role of RGD integrins in activating latent TGF‐β, we found that CWHM‐12 inhibited TGF‐β signaling and its pro‐fibrotic gene targets. However, our data also revealed significant effects on other biological processes that influence the development of kidney fibrosis and CKD progression (Figure 5). Reduced levels of intrarenal EGF mRNA and urinary EGF protein strongly correlated with interstitial fibrosis, estimated GFR, and rate of decline in kidney function in three independent patient cohorts (Ju et al., 2015). Consistent with this finding, *Egf* mRNA expression was reduced in our nephrotoxicity model. However, importantly, reduced expression was abrogated by CWHM‐12 treatment. In addition to being a biomarker for CKD, several studies support a functional role for EGF signaling in kidney fibrosis (Kok, Falke, Goldschmeding, & Nguyen, 2014; Liu et al., 2012; Lyu et al., 2018). A recent study identified a metabolic switch from oxidative phosphorylation to glycolysis associated with tubulointerstitial fibrosis and progressive CKD in human kidneys (Lemos et al., 2018). These data indicated that this process was driven by innate immune signals and *Myc*‐dependent gene transcription. Our results revealed that CWHM‐12 reduced gene expression associated with innate immunity and partially restored expression of genes associated with oxidative phosphorylation in injured kidneys. Moreover, CWHM‐12 also reduced *Myc* expression by 35%, suggesting its protective effect may be mediated in part by reducing *Myc*‐dependent gene expression. Interestingly, c‐Myc has been reported to directly activate transcription of the gene encoding αv in renal fibroblasts, which in turn increased TGF‐β activation (Shen et al., 2017). Reduced *Myc* expression with CWHM‐12 treatment might thus account in part for the observed reduction in integrin subunit expression.

While all αv integrins are formally capable of activating latent TGF‐β by direct binding, they may also have TGF‐β‐independent actions on ECM interactions and vasculature that support fibrosis (Babic, Chen, & Lau, 1999; Gao, 2006; McCurley et al., 2017; Parker et al., 2014). For example, in a rat ischemia‐reperfusion injury model, specific antibody neutralization of αvβ5 diminished acute renal damage, which was correlated with decreased kidney pericyte adhesion, migration, and vascular permeability (McCurley et al., 2017). The pathway analysis of global gene expression also uncovered highly significant changes in ECM organization and collagen metabolic processes. Independent of the direct biochemical role in TGF‐β activation, RGD‐binding integrins are important mechanosensors and transducers that provide cells such as myofibroblasts and their precursors’ information regarding the components and stiffness of their local ECM (Fiore et al., 2018; Lampi & Reinhart‐King, 2018; Santos & Lagares, 2018). There is evidence that fibrotic ECM adapts cells to a pro‐fibrotic phenotype rather than vice versa (Nagarajan et al., 2007). Furthermore, the RGD‐containing protein fibronectin, which is bound and assembled by integrin α5β1, is robustly deposited in remodeling tissue and is essential to collagen matrix deposition and maintenance (Zollinger & Smith, 2017). It was recently reported that senescent fibroblasts secrete high numbers of extracellular vesicles carrying fibronectin that engage α5β1 to confer an invasive phenotype on recipient fibroblasts (Chanda et al., 2019). Thus, interference with cellular signaling from fibrotic matrix molecules represents other mechanisms by which integrin antagonists may mitigate myofibroblast differentiation, migration, function, and survival. Our RNA‐seq data revealed that CWHM‐12 treatment downregulated genes that mediate fibronectin binding (*p* = 1.37 × 10^–5^).

In conclusion, our preclinical study showed that small‐molecule RGD peptidomimetic antagonists that target several pro‐fibrotic integrins, including one that was administered orally, ameliorated fibrosis and improved renal function in a model of nephrotoxicity. Unlike strategies that globally inhibit TGF‐β, which tend to be limited by side effects, this approach may be better tolerated due to the unique temporal and spatial characteristics of integrin expression at sites of injury. The strong correlation of our gene expression profiling with studies in humans further supports the clinical translational potential of our studies. To advance RGD integrin antagonists to the clinic, future investigation will need to determine whether they are effective when given later in the course of injury using varied dosing regimens and in different injury models. Safety will also need to be confirmed in detailed toxicology studies.

## CONFLICT OF INTEREST

All authors have read the journals' policy on disclosure of potential conflicts of interest. Rauchman, Basta, Robbins, Stout, and Prinsen have no conflicts of interest to declare. Griggs owns intellectual property rights and stock in, received grants from, and consults for Indalo Therapeutics.
