# Supplementary material for: Pharmacologic inhibition of RGD‐binding integrins ameliorates fibrosis and improves function following kidney injury
Source: Physiol Rep. 2020 Apr 13;8(7):e14329. doi: 10.14814/phy2.14329 (PMC7153038; doi:10.14814/phy2.14329)
Supplement: Supplementary file 1 [file PHY2-8-e14329-s001.pdf]

Supplementary Table 1.

CWHM-12 plasma concentrations (ng/mL) following 100 mg/kg/day mini-pump implantation of CWHM-12 to mice				
Mouse #	Kidney treatment	Day		
		0	5	13-27
21	PBS Uninjured	3878	7157	906
22		6906	5549	1213
23		570	560	2559
24		12318	13717	1028
25		3293	2317	1807
	**Mean**	**5393**	**5860**	**1503**
	**SD**	**4478**	**5103**	**684**
26	AA Injured	5914	4571	1064
27		5852	3769	ns
28		3317	4376	1649
29		16733	3624	1034
30		4423	2499	5453
31		2688	11156	ns
32		5964	2721	ns
33		3613	3659	2653
34		1877	4480	2108
35		5257	2081	ns
36		8682	2923	4430
37		3069	4765	3075
38		4745	4086	4724
39		4189	6508	4280
40		6754	13510	4157
	**Mean**	**5538**	**4982**	**na**
	**SD**	**3557**	**3205**	**na**

Drug levels were below the lower limit of detection (<1 ng/ml) for all vehicle treated animals; ns-no sample; na-not applicable because samples were obtained on different days. Uninjured animals #21-25 received an IP injection of PBS 1 day (day 0) after osmotic mini-pump insertion. Injured animals #26-40 received an IP injection of Aristolochic acid (AA) on day 0.

Supplementary Table 2.

CWHM-680 plasma concentrations (ng/mL) 24 hours post-dose following oral administration of CWHM-680 at 100 mg/kg/day (QD) to mice for 23 days				
Mouse #	Kidney treatment	Day		
		1	7	23
101	PBS Uninjured	36	103	373
102		19	152	310
103		153	22	38
104		83	ns	ns
105		969	74	41
	**Mean**	**252**	**88**	**191**
	**SD**	**404**	**54**	**176**
106	AA Injured	585	385	884
107		42	63	631
108		302	639	8770
109		21	218	1251
110		653	823	4629
111		108	111	423
112		1228	631	ns
113		1596	2178	2589
114		67	452	2243
115		49	25	179
116		45	49	1439
117		40	44	1174
118		1011	91	1803
119		22	23	2011
120		10	1231	3879
	**Mean**	**385**	**464**	**2279**
	**SD**	**517**	**594**	**2255**

Drug levels were below the lower limit of detection (<1 ng/ml) for all vehicle treated animals; ns-no sample. Uninjured animals #101-105 received an IP injection of PBS 1 day (day 0) after oral administration of CWHM-680. Injured animals #106-120 received an IP injection of Aristolochic acid (AA) on day 0.
